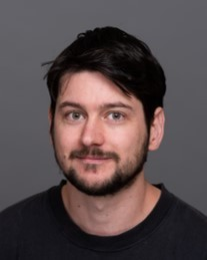# Combining PARP Inhibition with Platinum, Ruthenium or Gold Complexes for Cancer Therapy

**DOI:** 10.1002/cmdc.202000391

**Published:** 2020-09-10

**Authors:** Nur Aininie Yusoh, Haslina Ahmad, Martin R. Gill

**Affiliations:** ^1^ Department of Chemistry Faculty of Science Universiti Putra Malaysia 43400 UPM Serdang, Selangor Malaysia; ^2^ Integrated Chemical Biophysics Faculty of Science Universiti Putra Malaysia 43400 UPM Serdang, Selangor Malaysia; ^3^ Department of Chemistry Swansea University Swansea Wales (UK

**Keywords:** ruthenium, platinum drugs, PARP inhibitors, cancer, combination therapy

## Abstract

Platinum drugs are heavily used first‐line chemotherapeutic agents for many solid tumours and have stimulated substantial interest in the biological activity of DNA‐binding metal complexes. These complexes generate DNA lesions which trigger the activation of DNA damage response (DDR) pathways that are essential to maintain genomic integrity. Cancer cells exploit this intrinsic DNA repair network to counteract many types of chemotherapies. Now, advances in the molecular biology of cancer has paved the way for the combination of DDR inhibitors such as poly (ADP‐ribose) polymerase (PARP) inhibitors (PARPi) and agents that induce high levels of DNA replication stress or single‐strand break damage for synergistic cancer cell killing. In this review, we summarise early‐stage, preclinical and clinical findings exploring platinum and emerging ruthenium anti‐cancer complexes alongside PARPi in combination therapy for cancer and also describe emerging work on the ability of ruthenium and gold complexes to directly inhibit PARP activity.

## Graphical abstract

**Resistance is futile**: This review summarises cellular, preclinical and clinical work on the rational combination therapies between platinum, ruthenium and gold‐based complexes with‐and as‐ inhibitors of PARP, a key DNA repair enzyme. Given the impressive clinical findings to date, this combination strategy may be effective in modern cancer treatment.

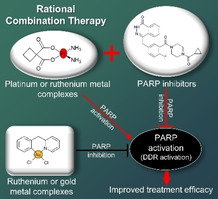

## 1 Introduction

Cancer remains one of the primary causes of death with a high number of global incidences reported annually. For example, in 2018, 18.1 million new cancer cases and 9.6 million cancer‐related deaths were reported.[^1^, ^2^] It is predicted that these numbers will rise within the next two decades. Currently, the routine methods for cancer treatment are surgical resection or radiotherapy alongside periods of chemotherapy.^[3]^ Despite having a high success rate, the efficacy of these strategies is limited by various factors such as the mass of the tumour to be removed, the stage of tumour progression, the availability (and affordability) of radiotherapy, the occurrence of metastatic tumours and the patient's health status.^[4]^ As a result, chemotherapy remains the most common and realistic option for cancer treatment.

Rational combination therapies of drugs that act on multiple targets and pathways are being sought to overcome the limited clinical options available within conventional chemotherapy.[^5^, ^6^] If such a drug combination results in additivity or synergy (a total effect greater than the sum of the individual effects of each drug), this has the distinct advantage that lower therapeutic doses of each individual drug can be used compared to each drug administered as a single‐agent.[^7^, ^8^, ^9^] Furthermore, considering the heterogenous nature of many cancers, drug combinations have potential in reducing the emergence of drug resistance and chance of relapse.[^10^, ^11^] Although a drug combination can give synergistic or additive interactions in cancer cells, the combination needs to have a high level of selectivity or large therapeutic index (TI), which is typically referred to toxicity to cancer cells over normal cells. Finally, in developing combinations of several chemotherapeutic agents, overlapping toxicity needs to be considered, especially with regard to the drug doses and scheduling.^[12]^

Cellular DNA is constantly being subjected to various endogenous and environmental damages. If the damage burden is high, this can interfere with fundamental cellular processes and cells will ultimately undergo cell death. Therefore, cells have evolved numerous DNA damage response (DDR) signalling networks to ensure genomic stability and to sustain continuous cellular progression and growth.^[13]^ Combined with their high rate of replication and inherent genomic instability, DDR defects are one of the traditional hallmarks of cancer.^[14]^ The subsequent development of highly specific DDR inhibitors along with the concept of synthetic lethality has led to a paradigm shift in small molecule‐based cancer therapy.^[15]^ Arguably the most successful example of this are inhibitors of poly (ADP‐ribose) polymerase (PARP), one of the key DNA repair enzymes in DDR signalling pathways.^[16]^ PARPs have become the rational targets in anti‐cancer drug research for the development of new drugs particularly for ovarian and breast cancers with defective breast cancer susceptibility gene (BRCA).[^17^, ^18^, ^19^] Several PARP inhibitors (PARPi) have progressed to clinical trials, and the PARPi olaparib (Lynparza®) has been approved for clinical treatment of BRCA‐mutated HER2‐negative metastatic breast cancer (2018), gBRCAm metastatic pancreatic cancer (2019) and maintenance of BRCA‐mutated (gBRCAm or sBRCAm) advanced epithelial ovarian cancers (2018).

While improved therapeutic response to PARP inhibition in BRCA1/2 mutated‐cancers has been shown, PARPi treatment inherently exerts limited efficacy in the treatment of cancers without homologous recombination (HR) deficiency.^[13]^ Considering BRCA‐deficient cancers are a relatively small subset of total cases, chemical strategies to extend the use of PARPi to a wider range of cancers are under investigation.^[20]^ Numerous studies in early‐stage, preclinical and clinical studies have now been conducted to examine a wider use of PARPi in combination therapy alongside various DNA‐damaging therapeutics in BRCA1/2‐proficient cancers.[^21^, ^22^] This aims to take advantage of greater understanding of DDR signalling in response to DNA damage and utilise the benefits that combination therapy offers. The fact several PARPi are FDA‐approved then makes this research clinically translatable.

With the discovery of the platinum‐based drug cisplatin by Barnett Rosenberg and co‐workers in 1960, a milestone in the history of metal‐based complexes in treating cancers was witnessed.^[23]^ Briefly, cisplatin induces platinum‐DNA adducts that cause blockage of replication fork progression, ultimately resulting in unrepairable DNA damage in the form of cytotoxic DNA double‐strand breaks (DSBs).[^24^, ^25^] Based on the success of cisplatin, inorganic medicinal chemists have since examined alternative transition metal centres such as ruthenium, palladium or rhodium to design complexes which target highly proliferative cancerous cells with improved therapeutic indices compared to cisplatin.[^26^, ^27^] A wide range of metal‐based complexes have been studied as single agents towards cancer cell lines and their efficacy, mechanism of actions and, in some cases, toxicity have been elucidated.[^28^, ^29^, ^30^] However, as is the case for the majority of anti‐cancer drugs, treatment with a single agent may not lead to sufficient tumour suppression to improve disease outcome or patient survival.[^11^, ^13^]

In this review, we discuss the various ongoing and completed studies that are examining metal‐based complexes for cancer therapy, with a particular focus on the rational combination of DNA‐targeting complexes alongside PARPi, with the aim of achieving additive or supra‐additive (synergistic) cancer cell killing, leaving non‐malignant cells unharmed and ultimately improving disease outcomes.

## 2 Metal‐Based Complexes as DNA‐Damaging Agents

Inhibiting DNA synthesis remains one of the central strategies in cancer therapy.^[31]^ This is based on the principles that cancer cells possess higher proliferation rates than the majority of normal cells and are more sensitive to certain forms of DNA damage due to their inherent genomic instability.[^22^, ^32^] Typically, the anti‐cancer activities of newly found or synthesised compounds are explored by determining DNA‐drug interactions in cell‐free conditions.^[33]^ In cells, these agents generate DNA damage by several mechanisms such as modifying the chemical structure of DNA bases, the generation of DNA crosslinks or adducts, replication fork stalling, or oxidative stress, all of which might potentially lead to DNA damage and ultimately cell death if damage persists.^[34]^

### 2.1 Platinum metal‐based complexes

**Figure 1:**
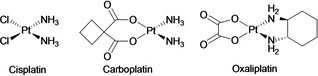
Structures of platinum‐based anti‐cancer drugs.

Platinum metal‐based drugs have become the standard first‐line chemotherapy for solid tumours and are largely employed in chemotherapy regimens (Figure 1).[^35^, ^36^] Among them, cisplatin (cis‐diamminedichloroplatinum(II)) was the first metal‐chemotherapeutic approved in 1978 and is, to date, part of the standard drug‐used in chemotherapy against a number of cancer types, including head and neck, testicular, cervical, oesophageal, ovarian and small cell lung cancers.^[25]^ Cisplatin induces inter‐ and intra‐strand platinum‐DNA crosslinks resulting in blockage of replication fork progression. This damage does not necessarily lead to immediate cytotoxic impact, but rather impacts cell‐cycle progression and induces cell‐cycle arrest. However, if stalled forks cannot be restarted, the resultant DSB DNA damage generated by replication fork collapse then leads to mitotic catastrophe and/or apoptosis.^[35]^ After the breakthrough of cisplatin, the second‐ and third‐generation cisplatin analogues, carboplatin (cis‐diammine(1,1‐cyclobutanecarboxylato) platinum(II)) and oxaliplatin (1R,2R‐diaminocyclohexane oxalatoplatinum(II)) were developed to reduce toxicity to tissues or organs, improve chemical stability and expand the scope of activity of these platinum compounds.[^37^, ^38^] Both agents successfully passed phase III clinical trials and are now FDA‐approved anti‐cancer drugs. Likewise, they react with DNA forming platinum‐induced intra‐ and inter‐strand crosslinks and were proven effective in various cancers with reduced toxicity compared to cisplatin.[^23^, ^39^]

Despite their clinical success, these platinum drugs possess inherent clinical drawbacks such as high general toxicity even when administered at lower doses, and poor selectivity against normal cells leading to severe adverse effects including myelosuppression, nephrotoxicity and neurotoxicity.^[25]^ This class of drug has also proven to be ineffective in patients with intrinsic or acquired chemoresistance.[^40^, ^41^, ^42^] These well‐established limits can significantly reduce their efficacy during treatment or even render them ineffective.[^43^, ^44^] Galuzzi *et al*. classified the mechanisms of resistance towards cisplatin into four main categories: (a) “Pre‐target resistance”, by reducing the likelihood of cisplatin cellular accumulation, thereby preventing the interaction between cisplatin and DNA. This includes reducing drug uptake and increasing drug efflux and drug detoxification. (b) “On‐target resistance”, by reversing the effects of cisplatin‐induced DNA adducts. (c) “Post‐target resistance”, by enhancing DNA repair capability that is activated following cisplatin‐induced DNA lesions. (d) “Off‐target resistance”, by molecular mechanisms which are not directly associated with cisplatin induced signals but enable cells to circumvent cisplatin‐induced cell death.[^45^, ^46^] These clinical drawbacks in platinum drugs have encouraged substantial efforts to replace them with suitable alternatives by using other transition metal complexes with higher efficacy and lower systemic toxicities – i. e. improved therapeutic windows – in tumour treatment.[^47^, ^48^, ^49^, ^50^]

### 2.2 Ruthenium metal‐based complexes

Ruthenium metal complexes have attracted a great amount of interest in the last two decades for their anti‐cancer properties. Initially, complexes were designed to coordinately bind DNA via the ruthenium metal centre with similar substitution kinetics to platinum complexes but altered potency *in vitro* and *in vivo*.[^51^, ^52^] In addition to metal centre‐based reactivity, the coordinated ligand(s) can interact with DNA or protein targets through reversible or covalent binding mechanisms. Ndagi *et al*. provide a review describing how incorporating an octahedral ruthenium metal centre can bring a unique contribution to drug design.^[26]^

**Figure 2:**
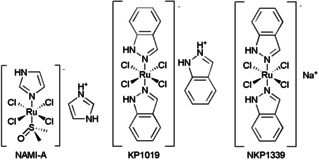
Structures of three clinical ruthenium complexes.

The first three ruthenium‐based complexes to successfully enter phase I clinical investigations were NAMI‐A, KP1019 and NKP1339 (Figure 2). NAMI‐A [ImH][*trans*‐RuCl_4_(DMSO)(Im)] where Im=imidazole and DMSO=dimethyl sulfoxide was the first ruthenium complex to enter clinical trials in 2002.^[53]^ Even though NAMI‐A showed low potency *in vitro* in terms of direct cytotoxicity on non‐small cell lung cancer (NSCLC) cells, it has effective and strong inhibitory efficacy on lung metastatic tumour *in vivo*.^[54]^ Subsequently, KP1019 [indazolium trans‐tetrachlorobis(1H‐indazole)ruthenate(III)] entered phase I clinical trial but failed to undergo further investigation due to its high hydrophobicity and poor water solubility limiting further clinical development.[^55^, ^56^] Hence, to improve the poor water solubility of KP1019, the derivative NKP1339 with improved aqueous solubility was developed and is currently undergoing clinical trials.[^57^, ^58^] In addition to improved transmembrane absorption efficiency, NKP1339 demonstrated disease stabilisation in a phase I study against solid tumours, most remarkably in patients with gastrointestinal neuroendocrine tumours.[^59^, ^60^] Reduced side effects were also noted in trial patients in the absence of clinical jaundice or other signs or symptoms.^[57]^

**Figure 3:**
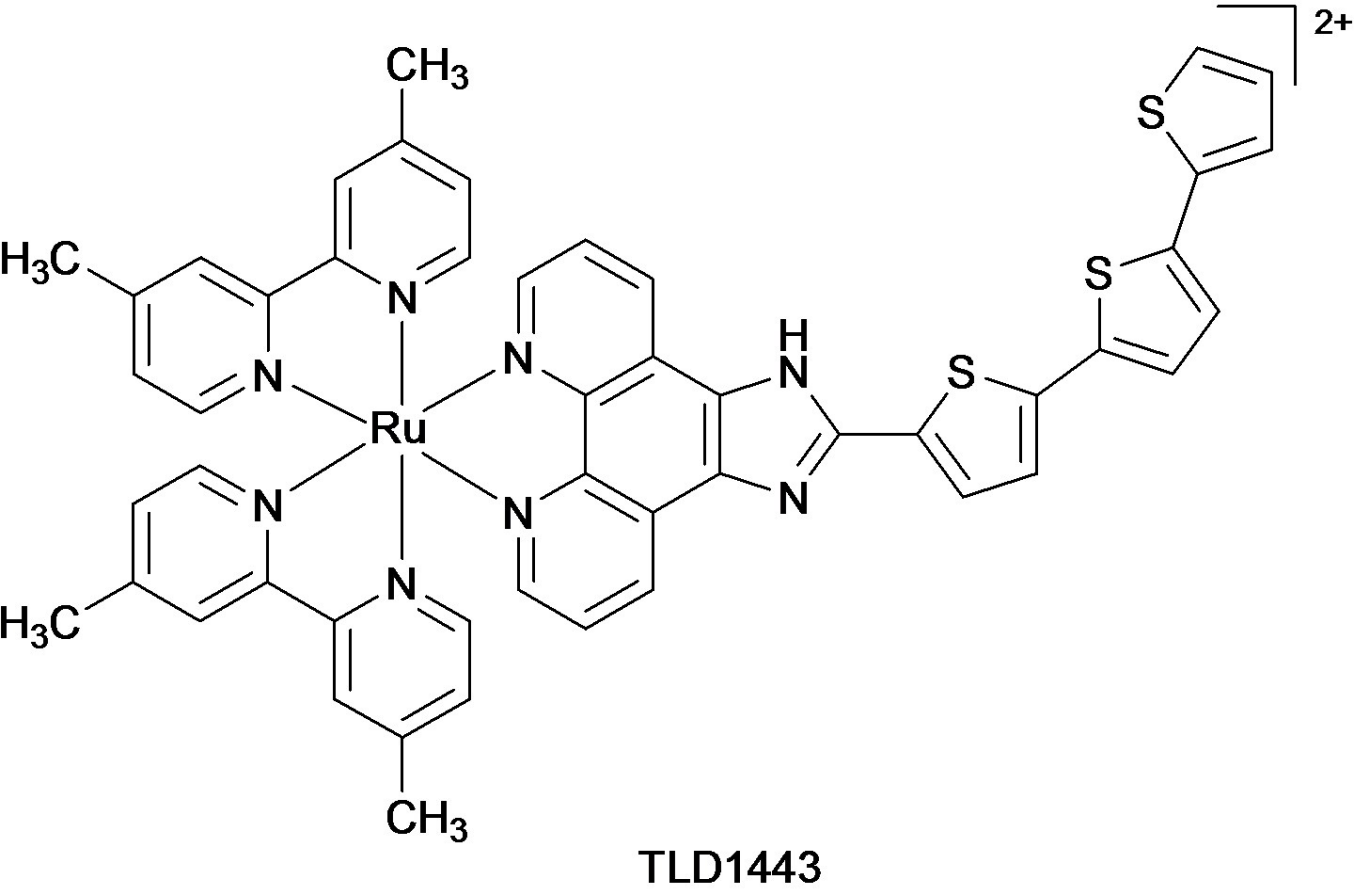
Structure of the RPC TLD1443.

Ruthenium(II) polypyridyl complexes (RPCs) have emerged as promising drug candidates due to their ability to form non‐covalent (reversible) interactions with DNA.[^61^, ^62^] DNA binding properties of these complexes can be tuned via substitution or modification of the ligand(s) around the Ru(II) centre.^[63]^ Many RPCs are phosphorescent and thus possess an imaging diagnostic capability that can be used to verify intracellular DNA or other biomolecule targeting.^[62]^ Finally, numerous RPCs act as photosensitizers for photodynamic therapy (PDT).[^64^, ^65^] In this latter capacity, the RPC photosensitizer TLD1443 [Ru(dmb)_2_(LL’)]^2+^ where dmb=4,4’‐dimethyl‐2,2’‐bipyridine and LL’=2‐((2’,2’’:5’’,2’’‘‐terthiophene)‐imidazo[4,5‐*f*][1,10]phenanthroline) was shown to significantly improve the efficacy of PDT and is currently undergoing phase II trials for bladder cancer patients (Figure 3).^[66]^

Other notable examples include the ruthenium(II)‐arene complexes which have attracted substantial interest in recent years following encouraging anti‐metastatic, anti‐angiogenic and anti‐tumoral properties *in vivo*.[^67^, ^68^] For example, RAPTA‐T Ru(η^6^‐toluene)‐(PTA)Cl_2_ and RAPTA‐C [Ru(η^6^‐**p**‐cymene)Cl_2_(PTA)] where PTA=1,3,5‐triaza‐7‐phosphaadamantane. Most encouragingly, RAPTA‐C appears to be well tolerated *in vivo* as determined by the high doses that can be tolerated in animals in comparison to platinum drugs. Similar to observations made on NAMI‐A, RAPTA‐C showed limited direct cytotoxicity on cancer cells **in vitro**; however, it exhibited strong anti‐metastatic behaviour **in vivo**.

Encouragingly, clinical findings demonstrated that the two ruthenium complexes of KP1019 and NKP1339 resulted in disease stabilisation and no severe adverse effects were noted.[^55^, ^57^] Compared to platinum drugs, they showed various clinical benefits including low general toxicity, greater tumour selectivity and importantly, potent efficacy on platinum‐resistant tumours was seen in a preclinical model.[^66^, ^69^] However, despite these major benefits, ruthenium complexes as single agents or monotherapy may not lead to sufficient tumour suppression, and the doses required for cancer cell killing are often very high. As discussed above, drug combination therapies are common in clinical practice and so using these therapeutic strategies to improve clinical response to ruthenium‐based complexes represents a promising line of research.

## 3 DNA Damage Response (DDR) Signalling Pathways

### 3.1 DNA repair mechanisms

DNA lesions at the base pair level such as alkylated nucleobases, single‐strand breaks (SSBs) or platinum‐associated intra‐ and inter‐strand crosslinks are repaired by base excision repair (BER), nucleotide‐excision repair (NER) and mismatch repair (MMR) pathways. Meanwhile large scale DNA lesions, such as DSBs and clustered damages require either homologous recombination (HR) repair or the non‐homologous end joining (NHEJ) repair pathways.[^13^, ^42^]

Small DNA adducts are mainly repaired by the BER pathway which requires specific DNA glycosylase to recognise mismatched base pairs in double‐stranded DNA and cleave the N‐glycosyl bond between the deoxyribose sugar and the nitrogenous base of the affected nucleotide, generating an abasic site.^[70]^ Apurinic/apyrimidinic (AP) endonucleases then create a nick in the phosphodiester backbone, and the resulting gap is filled by DNA polymerase, which is then sealed by DNA ligase. Another alternative DNA repair pathway involved in SSB repair that is functionally related to BER is the NER pathway with the major difference between these pathways is the size of damage that can be recognised.^[42]^ While BER detects non‐bulky DNA lesions and corrects damaged bases that are removed by a specific glycosylase, NER is particularly important to repair bulky DNA adducts. The major protein involved in NER includes excision repair cross‐complementation group 1 factor (ERCC1) which with ERCC4, forms a structure‐specific heterodimer complex of XPF‐ERCC1 endonuclease. This endonuclease cleaves the damaged strand on both sides of the lesion, and the oligonucleotide containing the lesion is excised. Finally, DNA polymerase fills the resulting gap by the process of HR. Most DSBs repair mechanisms are mediated by HR repair pathway with BRCA1/2 proteins serving as the critical components in the repair process.^[71]^ A crucial role of BRCA1/2 is binding the RAD51 protein, forming a complex on the DNA strand. Following this complex formation, the proteins that initiate the repair process are recruited to the damaged site. This process also involves a second, homologous intact strand of template DNA to allow for the precise restoration of the original DNA sequence.

### 3.2 DNA damage response (DDR) signalling

**Figure 4:**
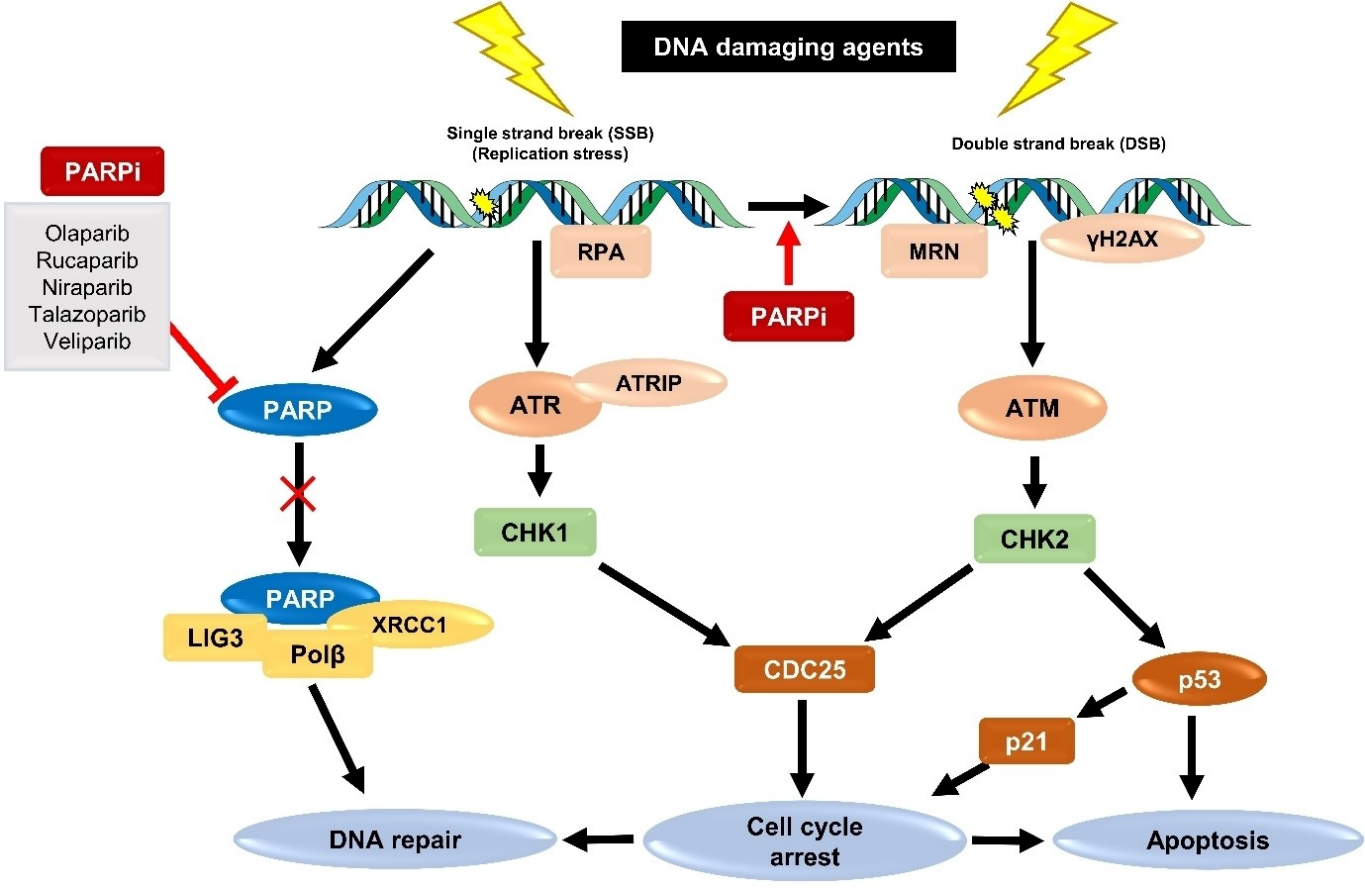
Simplified representation of the DNA damage response (DDR) signalling pathways.

The DNA damage response (DDR) signalling network is an intricate signal transduction cascade that prevents cell‐cycle continuation to allow the complete removal of DNA lesions prior to cell division (Figure 4).[^72^, ^73^] Three major DNA‐damage checkpoints have been described, located at G1/S, intra‐S, and G2/M phases of the cell‐cycle. The key signal transducers of downstream DDR pathways are ataxia telangiectasia mutated (ATM) and ataxia telangiectasia and Rad3 related (ATR) protein kinases. Upon DSB damage, ATM is directly recruited and activated by the MRN complex, a DSB‐recognising protein which phosphorylates a high number of DNA damage mediator proteins carrying the consensus sequence of ATM. This triggers the activation of downstream cell‐cycle regulator proteins, such as checkpoint kinase 2 (Chk2), which in turn activates downstream effector proteins that leads to cell‐cycle arrest and the activation of DNA repair pathways. This pathway also triggers the activation of p53, a tumour suppressor protein which decides cellular fate depending on repair efficiency or the level of DNA damage. When the level of damage is high, the intrinsic pathway of apoptosis is triggered, resulting cell death. Lesions due to stalled replication forks induce SSBs or DNA replication stress. In these cases, replication protein A (RPA) binds to the SSB and recruits ATR kinase via its association with ATR interacting protein (ATRIP). Finally, this activates the downstream regulator protein, checkpoint kinase 1 (Chk1), which in turn activates downstream effector proteins.

### 3.3 PARP: a key DDR enzyme family

PARPs are a family of 17 enzymes encoded by genes that mediate several cellular processes, including DNA damage repair, maintenance of genomic stability and regulation of transcriptional control.[^74^, ^75^] The role of PARP in DDR signalling was first reported in 1979, where PARP activity was found to increase after administration of chemo or radiotherapy.^[76]^ PARP activation is now known to be an early response to SSB repair.^[77]^ Alongside its role in SSB repair, PARP also mediates replication restart following replication fork stalling to ensure faithful genome duplication.[^78^, ^79^] PARP activation has been implicated to increased activities of BER and NER pathways, the predominant SSBs repair pathways in cells with a fully functional DDR capacity.^[80]^ At the molecular level, PARP is recruited and binds to SSBs through its highly conserved N‐terminal zinc finger domains, which in turn activates its C‐terminal motif leading to the hydrolyzation of NAD+. This generates long chains of ADP‐ribose monomers (PARylation) and subsequently initiates the repair process by actively recruiting other repair proteins, including X‐ray repair cross complementing protein 1 (XRCC1), DNA polymerase β (Polβ) and DNA ligase III (LIG3).^[81]^ Deletion of the PARP gene in experimental cell models alongside high levels of SSBs resulted in the accumulation of DSBs, cell‐cycle arrest and/or cell death.[^82^, ^83^]

### 3.4 PARP inhibitors

**Figure 5:**
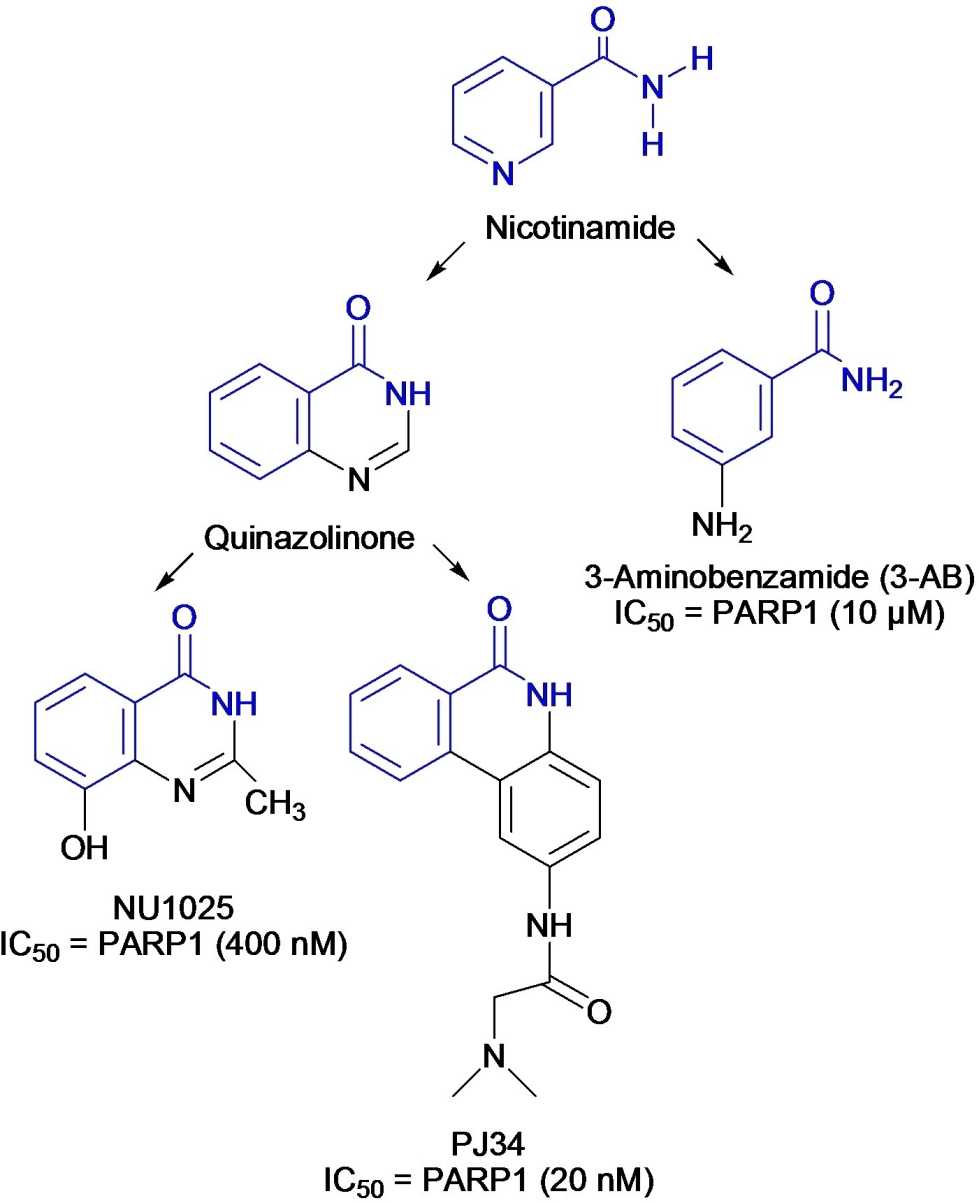
First‐generation PARP inhibitor, 3‐AB and second‐generation PARP inhibitors, PJ34 and NU1025. Nicotinamide/benzamide pharmacophore group shown in blue.

First‐generation PARPi were developed over 40 years ago and were simple analogues of nicotinamide such as 3‐substituted benzamides (*e. g*. 3‐aminobenzamide (3‐AB); Figure 5).^[84]^ Bernges and Zeller reported that by inhibiting PARP activity with 3‐AB (PARP1 half‐inhibitory concentration, IC_50_,=10 μM), the alkylating agent carmustine exhibited increased cytotoxicity in ovarian cancer cell lines.^[85]^ Studies conducted in pancreatic cancer cells by Jacob *et al*. similarly reported that inhibition of PARP activity using 3‐AB leads to improved responses to standard gemcitabine regimen.^[86]^ Although 3‐AB showed encouraging results in sensitising cells to genotoxic agents, high dosages were required in preclinical models. Second‐generation PARPi were subsequently developed in the 1990s based on quinazoline analogues (*e. g*. PJ34 and NU1025; Figure 5). These agents showed more efficient targeting and substantially improved activity in inhibiting purified human PARP1 enzyme than the early‐generation PARPi 3‐AB (PARP1 IC_50_s=20 and 400 nM for PJ34 and NU1025, respectively).^[87]^

**Figure 6:**
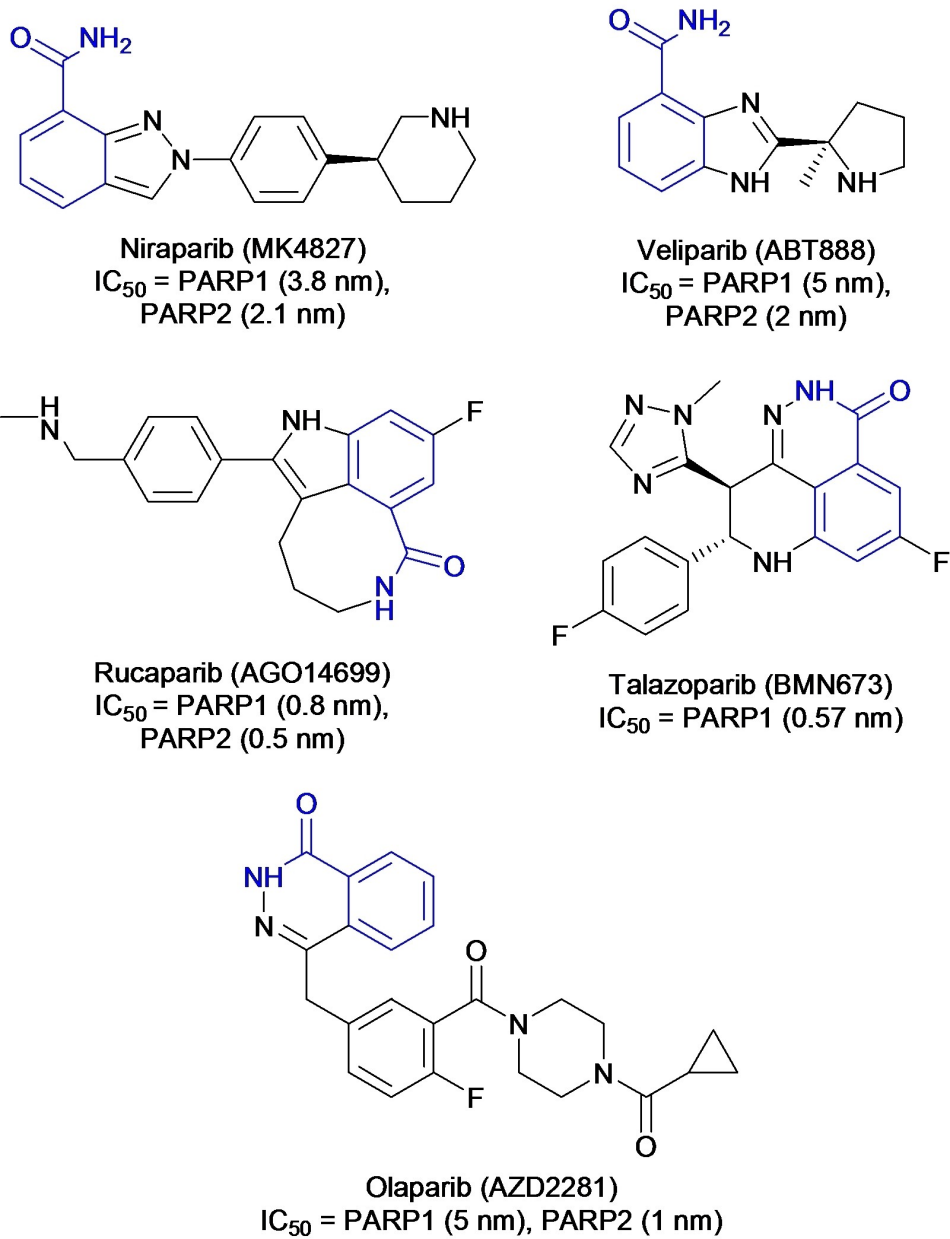
Structures of clinical and FDA‐approved PARP inhibitors with nicotinamide/benzamide pharmacophore group shown in blue

The breakthrough in this area was the discovery that PARPi act as a potent therapeutic agent in cancers harbouring HR repair defects, such as those with BRCA mutations.[^12^, ^88^, ^89^, ^90^] The basis for this synthetic lethality is that in HR‐deficient cells, DSBs generated by PARP inhibition cannot be repaired leading to mitotic catastrophe and/or apoptosis. Cells with normal HR function are able to repair PARPi‐induced DSB damage, resulting in synthetic lethality exclusively in HR‐deficient cells. Shortly after this realisation, further development of the third‐generation PARPi based on benzimidazoles led to more potent PARPi which rapidly entered clinical trials. Prominent examples include olaparib (AZD2281), rucaparib (AGO14699), niraparib (MK4827), veliparib (ABT888), and talazoparib (BMN673) (Figure 6).^[16]^ In 2015, olaparib (Lynparza®) and rucaparib (Rubraca®) have passed phase III clinical trials and are FDA‐approved for the treatment of advanced ovarian cancer patients with mutations in the germline BRCA1/2 genes.^[91]^ Niraparib (Zejula®) was additionally approved for the maintenance treatment of recurrent and platinum‐sensitive ovarian cancer patients. Most recently, talazoparib (Talzenna®) was approved for advanced and metastatic breast cancer patients.

Cellular investigations using these third‐generation inhibitors led to the discovery that PARPi can also trap PARP enzymes (termed as “PARP trapping”) at the damage site.[^92^, ^93^, ^94^] Trapped PARP‐DNA complexes have been suggested to be more cytotoxic than unrepaired SSBs caused by PARP inactivation.^[95]^ As a result, PARPi are often classed by their PARP trapping potency as well as PARP inhibitory effects.

Although breast and ovarian cancers with mutations in BRCA1/2 demonstrate exquisite sensitivity to PARPi, these cancers represent a relatively small subset of cancers.^[96]^ However, niraparib (Zejula®) also showed significant benefit in patients with functional HR repair capability, indicating that there are likely potential biomarkers and mechanisms that may provide sensitivity towards PARP inhibition in addition to HR pathway genes.^[12]^

### 3.5 Alterations in PARP: a mechanism of platinum drug resistance?

The activity of PARP in DNA repair has been shown to counteract many types of DNA damaging chemotherapies and thus is crucial for the emergence of resistance during prolonged cancer treatment.^[22]^ Interestingly, overexpression of PARP has been noted in several cancer cell lines compared to their normal counterparts.^[97]^ The role of PARP hyperactivation in therapeutic resistance to cisplatin in the majority of advanced tumours and human cisplatin‐resistant cancer cells has been described in detail by Michels *et al*.^[98]^ For example, NSCLC cells with poor responses to cisplatin treatment exhibited high expression level of PARP.^[99]^ Constitutive activity of PARP is also essential for treatment resistance and disease progression in glioblastoma‐initiating cells.[^100^, ^101^] A prominent example is provided by mechanistic studies by Lavrik *et al*. where the authors found that PARP is immediately activated in cells following the addition of platinum drugs. When PARP auto‐PARylates, BER pathway proteins are recruited to the DNA damage sites. While PARP is stabilised on the DNA strand, the recruitment of other BER proteins is hampered, hindering DNA repair processes.^[102]^

In addition to the role of PARP hyperactivation in platinum resistance, Amable *et al*. showed the association between high levels of ERCC1 expression and cisplatin resistance in cancer therapy.^[40]^ The basis for this is that increased ERCC1 expression levels is associated with increased NER repair capacity which effectively reverse the effects of platinum‐DNA adducts. These studies included data from ovarian, cervical, lung, liver, and gastric cancers, indicating that high level of ERCC1 expression could be an early potential indicator in determining cellular platinum resistance and disease progression.^[103]^ On the other hand, testicular, primary gastric, and lung cancer that have low levels of ERCC1‐XPF expression have been shown to be highly responsive to platinum drugs.^[42]^ In these cancers, improved survival of patients treated with platinum drugs was observed due to the reduced cells capability to repair DNA lesions, eliciting the importance of ERCC1 expression levels in treatment resistance.

Considering the enhancement of DNA repair capability is one of the major contributions to resistance in cancer cells, it follows that simultaneous inhibition of PARP activity alongside DNA‐targeting platinum and ruthenium metal complexes could suppress chemoresistance and in addition to heightening sensitivity towards these DNA‐damaging metal complexes.

## 4 PARP Combination Therapy: A Promising Strategy

The rational combination of a DNA‐damaging agent alongside a PARP inhibitor has long been hypothesised to induce synergistic activity.[^104^, ^105^] More recently, it has also become apparent that this strategy can expand the use of PARPi to a greater population of cancer types, independent of BRCA status.^[106]^ Olaparib, for example, has been shown to synergistically improve the activity of ionising radiation (IR),[^107^, ^108^] gemcitabine,^[109]^ the alkylating agent temozolomide,^[110]^ and topoisomerase inhibitors such as doxorubicin^[111]^ and topotecan^[110]^ in various cancers irrespective of their BRCA status. However, many of the DNA‐damaging agents tested alongside PARPi to date are potent cytotoxics and generate high levels of DSB damage; two features which may have unfavourable mechanistic and toxicity overlap with PARPi. In the following sections, we discuss the early‐stage, preclinical and clinical work of the rational combinations between platinum and ruthenium metal‐based complexes and PARPi for enhanced cancer therapy, as well as potential applications of PARPi in combination with cell‐cycle inhibitors.

### 4.1 Combination of cisplatin and 3‐AB or PJ34

An early *in vitro* study showed that the combination of cisplatin and PARPi 3‐AB resulted in enhanced cell‐cycle arrest and apoptosis in cisplatin‐resistant ovarian tumour cells.^[85]^ Other examples assessed the combination of cisplatin with the second‐generation PARPi PJ34. Mechanistic investigations in triple‐negative breast cancer (TNBC) cells revealed that the synergistic combination was mediated by sustained DNA damage and inefficient NER repair triggering apoptosis.^[112]^ Similarly, in lung cancer cells, the combination significantly increased DNA damage foci, resulting in the loss of clonogenic potential of these cells and ultimately triggering apoptotic cell death.^[99]^ PJ34 enhanced the suppressive effects of cisplatin in a dose‐dependent manner in the growth of HepG2 liver cancer cells, accompanied with increased apoptosis.^[113]^ This combination was shown to inhibit the growth of HepG2 cell‐derived tumours in nude mice and is one of the earliest *in vivo* studies that provided evidence to support this combination strategy.

### 4.2 Combination of cisplatin and olaparib

**Figure 7:**
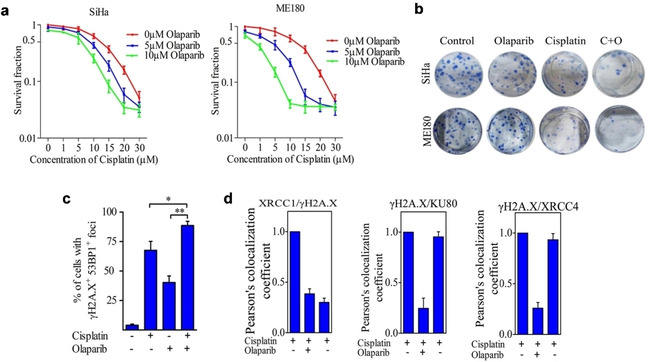
(a) Cell viability of cervical cancer SiHa and ME180 cells upon treatments with cisplatin and olaparib alone or in combination, as determined by MTT assay. (b) Clonogenic survival of cervical cancer cells upon treatments with cisplatin, olaparib or both. (c) Quantification of the percentage of cells showing γH2A.X+53BP1+cells as DNA damage marker upon treatments with the respective mentioned groups. (d) Quantification of relative co‐localisation of XRCC4/KU80/XRCC4 foci and γH2A.X foci in the respective mentioned groups. Figure adapted from reference 115 under the terms of the Creative Commons Attribution 4.0 International License (https://creativecommons.org/licenses/by/4.0/). Published by Springer Nature Limited. © The Author 2017.

The combination of cisplatin and olaparib in a small panel of lung cancer cell lines found cancer cell killing was achieved specifically in cells with low ERCC1 expression.^[114]^ Mechanistic studies indicated that this combination leads to sustained DSBs, prolonged G2/M cell‐cycle arrest and the activation of Chk1 signalling with a significantly marked increase in apoptosis. In cervical cancer cells, the combination of cisplatin and olaparib synergised with a significant anti‐proliferative effect and loss in clonogenic survival compared to single agents alone.^[115]^ These findings were accompanied with increased DSB levels and apoptotic cell death (Figure 7a–c). Further mechanistic investigations revealed that olaparib disrupts the localisation of the BER effector proteins XRCC1 and NHEJ proteins Ku80 and XRCC4 that modulate DNA repair efficiency in cells (Figure 7d). A phase I trial evaluating this combination showed improved tolerability and promising anti‐tumoral activity in patients with advanced ovarian, breast and other solid tumours, particularly those with BRCA1/2 mutations (Clinicaltrials.gov identifier: NCT00782574).^[116]^ At the end of the study, 41 % of the patients with measurable disease achieved overall objective response rate. Among patients who had ovarian and breast cancer with mutations in BRCA1/2, the overall objective response rate achieved were 43 % and 71 %, respectively.

### 4.3 Combination of carboplatin and olaparib

**Figure 8:**
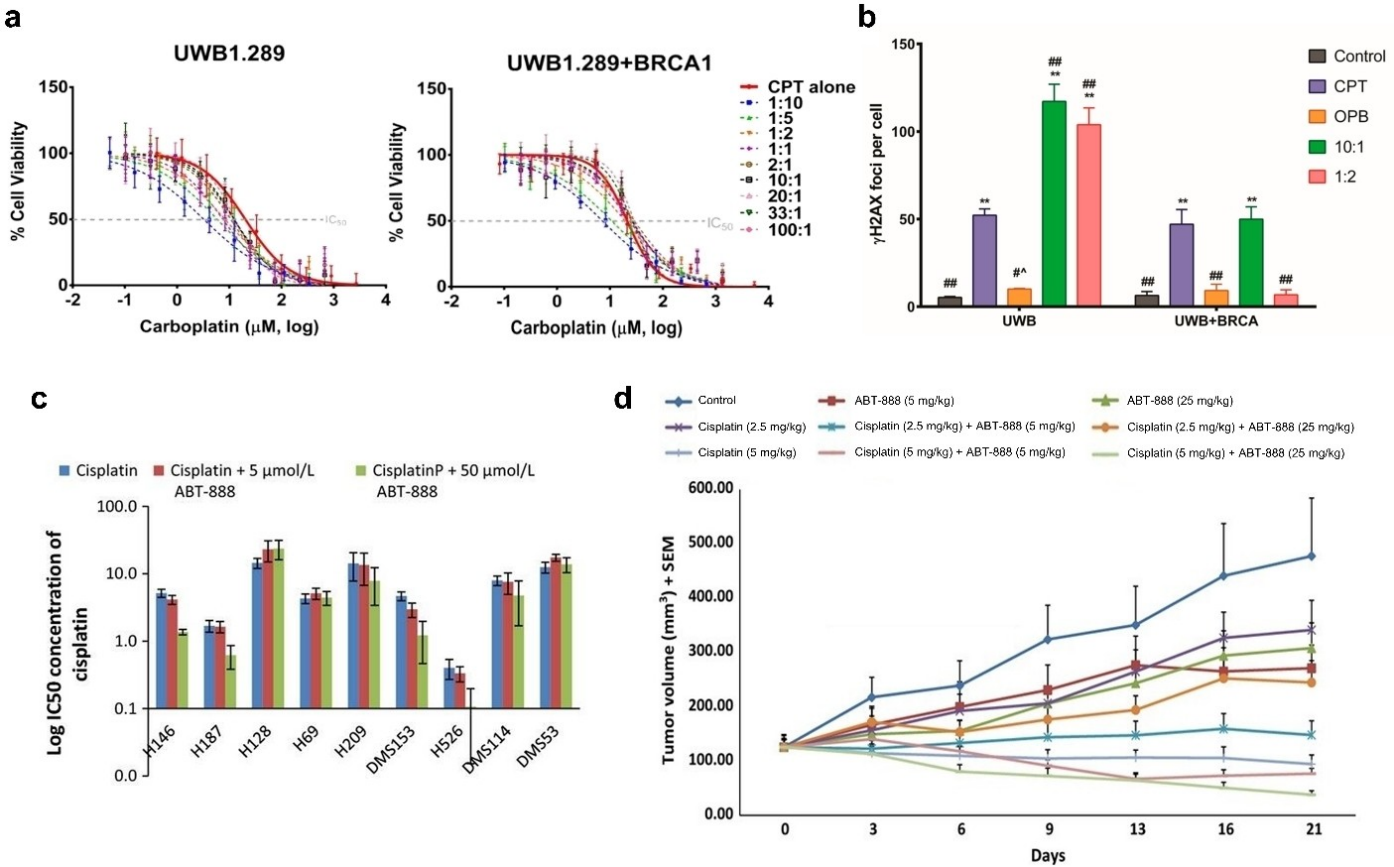
(a) Cell viability of BRCA‐deficient (UWB1.289) and BRCA‐proficient (UWB1.289+BRCA1) high grade serous ovarian cancer cells upon treatment with carboplatin (CPT) and olaparib (OPB) alone or in combination for 72 h, as determined by MTT assay. (b) Quantification of γH2AX foci formation as DNA damage marker upon treatments with carboplatin, olaparib or both for 72 h. (c) IC50 concentrations of cisplatin alone or in combination with 5 and 50 μmol/L of veliparib in nine different SCLC cells. (d) The tumour volume of H146 SCLC xenograft model following treatment with cisplatin and veliparib alone or in combination. Figure (a–b) reprinted (adapted) with permission from reference 119. Copyright 2018 American Chemical Society. Figure (c–d) adapted from reference 122 under the terms of the Creative Commons Attribution 3.0 Unported Licence (https://creativecommons.org/licenses/by/3.0/). Cancer Medicine published by John Wiley & Sons Ltd. © The Author 2014.

The concomitant administration of carboplatin and olaparib has been shown to induce lethality in a synergistic manner in MDA‐MB‐231 and CAL51 TNBC cell lines.^[117]^ This study included the phosphatidylinositol 3‐kinase (PI3K) inhibitor BKM12 as the P13K intracellular signalling pathway has become one of the molecular targets for cancer therapy due to its role in regulating the essential cellular functions, including metabolism, proliferation, cell survival, growth and angiogenesis.^[118]^ In addition to this, TNBC is characterised by extensive copy number alterations that promotes PI3K pathway activation with deficiencies in DNA damage HR repair. Drug synergy was observed in both TNBC cell lines with a clear impact on cell‐cycle progression. Western blotting and immunofluorescence studies indicated that this combination exerted its cytotoxicity via DNA damage, enhancing NHEJ repair while inhibiting HR repair. Another notable example includes a study on the combination of carboplatin and olaparib conducted in high‐grade serous ovarian cancer cells (HGSOC).^[119]^ Synergy was observed in both BRCA1/2‐proficient and ‐deficient cell lines, indicating that the therapeutic benefits of this combination is independent of BRCA status (Figure 8a). Mechanistic studies revealed that the synergy observed in BRCA‐deficient UWB1.289 cell lines were due to increased DNA DSBs (Figure 8b). This combination was also found to greatly inhibit the tumour growth of BRCA2‐mutated ovarian serous carcinoma.^[120]^

In a phase II clinical investigation, the combination of olaparib with carboplatin‐paclitaxel was investigated in high‐grade and recurrent ovarian cancer patients with sensitivity to platinum regimens and deficiency in BRCA genes (ClinicalTrials.gov identifier: NCT01081951).^[121]^ The combination significantly improved the chances of progression‐free survival; 9.6 months to 12.2 months in these patients, with the greatest clinical benefit observed in BRCA‐mutated patients. Importantly, an acceptable and manageable tolerability profile was reported with 12 of 81 patients (15 %) in the combination treatment group experienced serious adverse events compared to 16 of 75 patients (21 %) in the single‐agent treatment group. Collectively, this combination is considered safer and feasible than monotherapy especially for high‐grade and recurrent ovarian cancer patients.

### 4.4 Combination of carboplatin/cisplatin and veliparib

Despite showing limited single‐agent cytotoxicity, *in vitro* studies found the PARPi veliparib selectively potentiated the effects of cisplatin and carboplatin in five of nine small cell lung cancer (SCLC) cell lines (Figure 8c).^[122]^ Moreover, the combination of veliparib and cisplatin showed greater tumour growth inhibition compared to single‐agent treatment groups in a lung cancer xenograft model (Figure 8d). In addition to this early study, a phase I trial involving the combination of carboplatin and veliparib in advanced ovarian cancer patients was conducted (Clinicaltrials.gov identifier: NCT01063816).^[123]^ Overall, treatment with this combination was efficient and associated with an overall response rate of 49.2 %. In a recent phase III trial in 2018 involving six hundred and thirty‐four TNBC patients, the combination of carboplatin and veliparib with paclitaxel significantly improved the proportion of TNBC patients who achieved a pathological complete response compared to single agents, although the overall survival or progression free survival or was not conclusive (ClinicalTrials.gov identifier: NCT02032277).^[124]^ It was also reported that increased toxicities following the addition of carboplatin and veliparib was manageable and the treatment delivery of paclitaxel was not substantially affected.

### 4.5 Combining ruthenium‐based complexes with PARPi

Ruthenium metal‐based complexes have been examined extensively as single agents for their anti‐cancer properties.^[47]^ However, studies into the combination of ruthenium complexes with other compounds are uncommon due to several uncertain factors, including on how to select compounds for combination and the potential mechanism(s) of drug synergism.^[125]^ Nevertheless, several studies with first‐line anti‐cancer agents, including the combinations of Ru(II) complex/radiation,^[126]^ NAMI‐A/doxorubicin,^[127]^ RAPTA‐C/doxorubicin,^[67]^ Ru(II) complex/doxorubicin,^[128]^ NKP1339/sorafenib,^[129]^ and RAPTA‐C/erlotinib^[130]^ have been explored both *in vitro* and *in vivo*. Moreover, the combination of NAMI‐A/gemcitabine has been clinically investigated in phase I/II trials for NSCLC patients.^[131]^ Compared to single agents alone, favourable efficacies and synergistic anti‐tumour efficacy with desirable toxicity profiles were reported. However, studies on the combination of ruthenium‐based complexes with PARPi or other small molecules DDR‐targeting agents are a relatively new concept.

**Figure 9:**
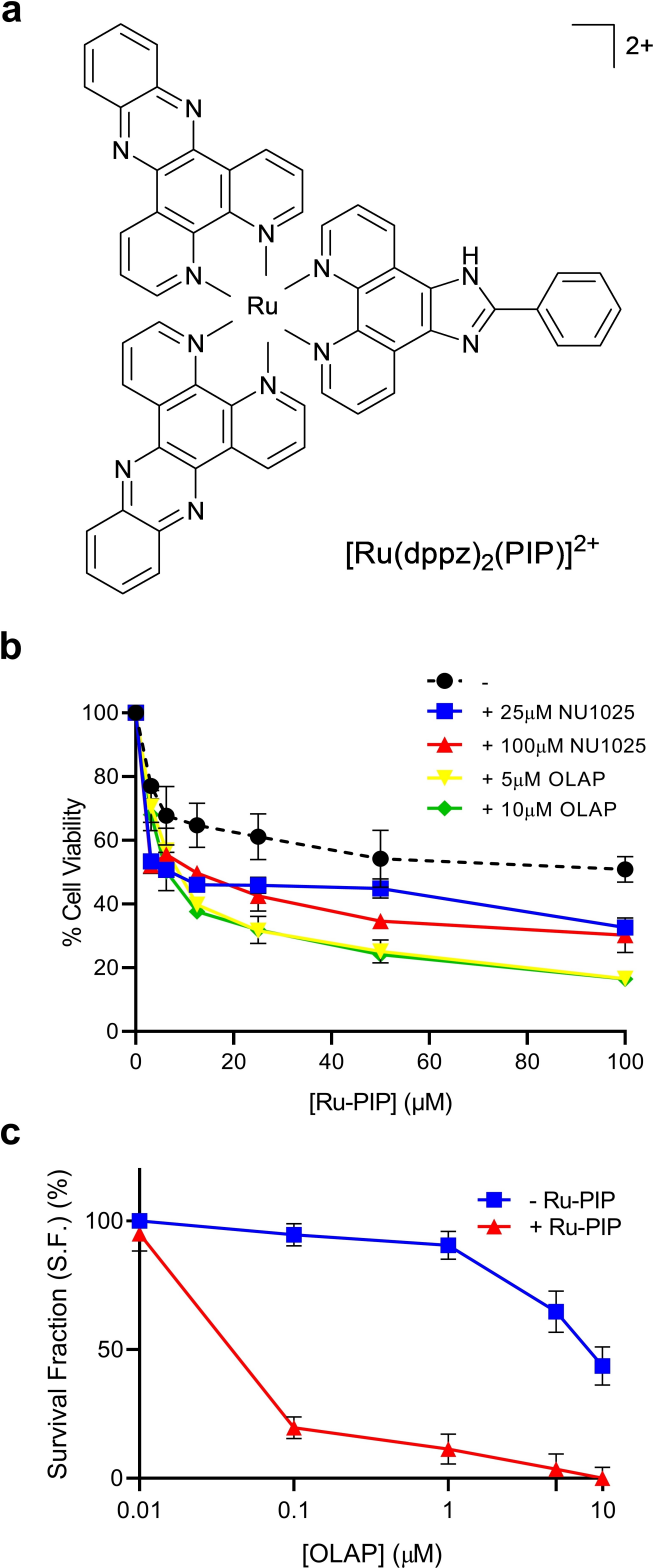
(a) Structure of [Ru(dppz)_2_(PIP)]^2+^ or Ru‐PIP. (b) Cell viability of BRCA wild‐type TNBC MDA‐MB‐231 cells following 24 h treatments with Ru‐PIP alone or in combination with PARP inhibitors (olaparib concentrations of 5 or 10 μM and NU1025 concentrations of 25 or 100 μM were used), as determined by MTT assay. (c) Clonogenic survival of MDA‐MB‐231 cells upon 24 h treatments with a concentration gradient of olaparib with and without Ru‐PIP (25 μM). Single‐agent Ru‐PIP has low impact on clonogenic survival at this concentration (S.F. >95 % at 25 μM), indicating it is sensitising TNBC cells to olaparib. Reprinted (adapted) with permission from reference 134. Copyright 2020 American Chemical Society.

Ruthenium(II) metallo‐intercalators have been shown to stall replication fork progression and generate high levels of replication stress in cancer cells.[^132^, ^133^] This finding provides justification for the assessment of this class of complexes alongside PARPi. Recently, we demonstrated that the combination of the multi‐intercalator [Ru(dppz)_2_(PIP)]^2+^ (or Ru‐PIP, Figure 9a) where dppz=dipyrido[3,2‐*a*:2′,3′‐*c*]phenazine and PIP=2‐(phenyl)‐imidazo[4,5‐*f*][1,10]phenanthroline with either NU1025, a second‐generation PARPi, or olaparib showed synergy in BRCA wild‐type TNBC breast cancer cells (Figure 9b).^[134]^ As the predominantly cytostatic Ru‐PIP acts to stalls replication fork progression *without* triggering a DSB damage response,^[133]^ PARPi‐mediated replication fork collapse results in a significant increase in DSBs damage, accompanied by G2/M cell cycle arrest and cell death via apoptosis. This combination led to a dramatic increase in the potency of olaparib, where 300‐fold greater activity due to the addition of Ru‐PIP was observed by clonogenic survival assay (Figure 9c). Promisingly, a mild impact on non‐malignant NHDF human fibroblast cells was observed, indicating a potential high tumour selectivity activity of this combination which merits further investigation *in vivo*.

While the combination of ruthenium with PARPi demonstrated good results, Gill *et al*. has also showed the potential of combination with other DDR inhibitors. The concurrent treatment of Ru‐PIP with a Chk1 inhibitor, CHIR‐124 showed synergistic apoptosis in HeLa cervical cancer cells, with a significant increase in DSBs resulting from stalled replication fork collapse while having minimal impact on non‐malignant HFF human epithelial cells.^[133]^

As discussed, PARP inhibition in combination therapy has mainly utilised potent cytotoxic DSB‐generating agents that activate G2/M arrest. It is therefore noteworthy that a cytostatic replication inhibitor and PARPi can also achieve synergy in cancer cells.

### 4.6 Combination of PARPi and cell‐cycle inhibitors

Synergy with PARPi is not necessarily limited to DNA‐damaging agents. Although PARPi are active in HR‐deficient cancers, particularly those lacking BRCA1/2, their utility is limited by the development of resistance which may occur through restoration of HR function.^[135]^ These BRCA‐deficient cells rely heavily on cell‐cycle regulators to reverse the effects of PARP inhibition and become PARPi‐resistant cells. As such, the combination of PARPi and cell‐cycle checkpoint inhibitors such as ATR,[^136^, ^137^, ^138^] Chk1,^[139]^ and WEE1[^140^, ^141^] have recently been investigated in several different cancers. These combinations demonstrated enhanced efficacy, especially in overcoming PARPi‐resistance and act to abrogate PARPi‐induced G2/M arrest and prevent DNA damage repair.

**Figure 10:**
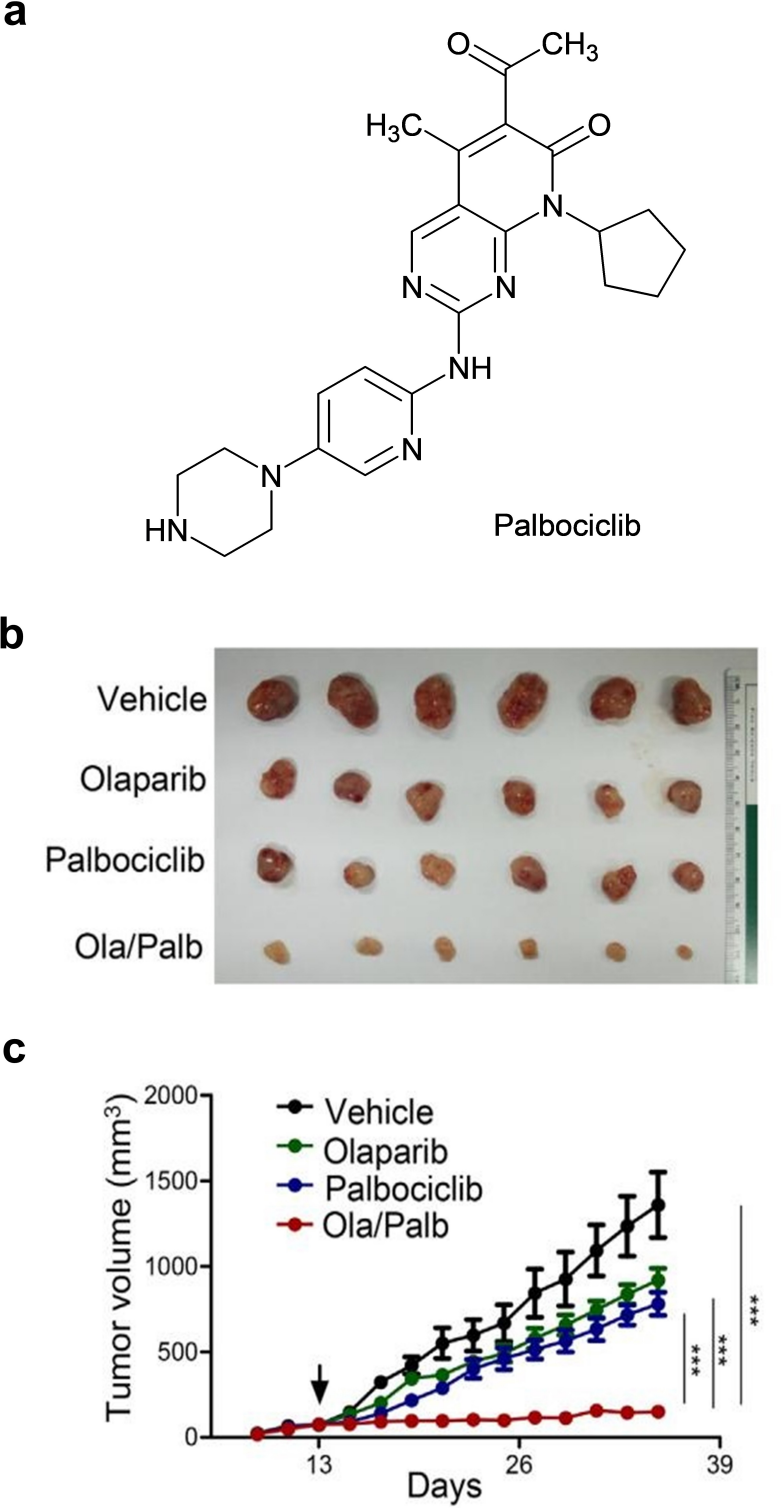
(a) Structure of palbociclib. (b) Representative images of ovarian cancer A2780 xenografted tumours isolated from mice following treatments with olaparib, palbociclib or both. (c) The tumour volume of A2780 xenografted mice in the respective mentioned groups, with the arrow indicating the starting date of the treatment. Reprinted from EBioMedicine, 43, Yi, J. et al., MYC Status as a Determinant of Synergistic Response to Olaparib and Palbociclib in Ovarian Cancer, 225–237, Copyright 2019, with permission from Elsevier.

Other potential combination partners for PARPi are inhibitors of cyclin dependent kinases (CDKs).[^142^, ^143^] In addition to playing a crucial role in cell‐cycle regulation, CDK1 was found to phosphorylate BRCA1, an event essential for efficient BRCA1 focus formation and HR repair.^[144]^ Therefore, CDK inhibition presents a method to induce a form of BRCA‐deficiency, thereby rendering treated cells sensitive to PARPi. Based upon this concept, a CDK1 inhibitor was found to sensitise BRCA‐proficient cancers to PARP inhibition in human lung NSCLC xenografts.^[144]^ Recent work with olaparib and palbociclib (a CDK4/6 inhibitor, Figure 10a) has demonstrated potent synergy in ovarian cancer cells overexpressing MYC both *in vitro* and *in vivo*.^[145]^ This combination significantly increased anti‐proliferative effects *in vitro* and inhibited tumour growth *in vivo* compared to single‐agent treatment groups (Figure 10b–c). Mechanistically, this combination induced HR deficiency in a MYC‐dependent manner. In another study, dinaciclib, a CDK1, 2, 5, 9 and 12 inhibitor diminished HR pathway function and reversed acquired PARPi‐resistance in BRCA‐proficient and ‐deficient TNBC.^[146]^ The combination of veliparib and dinaciclib against patients with advanced solid tumours who are not germline BRCA carriers was evaluated in a phase I study. This found that twenty‐four patients (38 %) had stable disease as the best response with nine progression‐free (ClinicalTrials.gov identifier: NCT01434316).^[147]^ These findings show the potential of using this combination strategy to re‐establish PARPi sensitivity. Bearing in mind many anti‐cancer transition metal complexes achieve potent effects on cell‐cycle progression without generating cytotoxic or genotoxic DNA damage, this may be a lucrative area for future investigation.

## 5 Metal Complexes as PARP Inhibitors

In addition to their use in combination therapy alongside organic PARP inhibitors, the metal complex itself can inhibit PARP activity. Here, we summarise work that has explored this emerging concept.

### 5.1 Ruthenium complex as PARP inhibitors

A small number of studies have explored the ability of ruthenium complexes to function as PARP inhibitors. Mendes *et al*. showed that RAPTA‐T and NAMI‐A were found to be more effective than 3‐AB in inhibiting purified human PARP1 enzyme (24 h IC_50_ concentrations of 28 μM and 18.9 μM for RAPTA‐T and NAMI‐A, respectively, compared to 3‐AB with IC_50_=33 μM).^[148]^ An organometallic ruthenium(II) complex, [Ru^II^Cp(bipy)(PPh_3_)][CF_3_SO_3_] (or TM34) (bipy=2,2′‐bipyridine and PPh_3_=triphenylphosphine) demonstrated the strongest PARP1 inhibitory activity reported so far for a ruthenium complex, with a 24 h IC_50_ value of 1 μM.^[149]^

**Figure 11:**
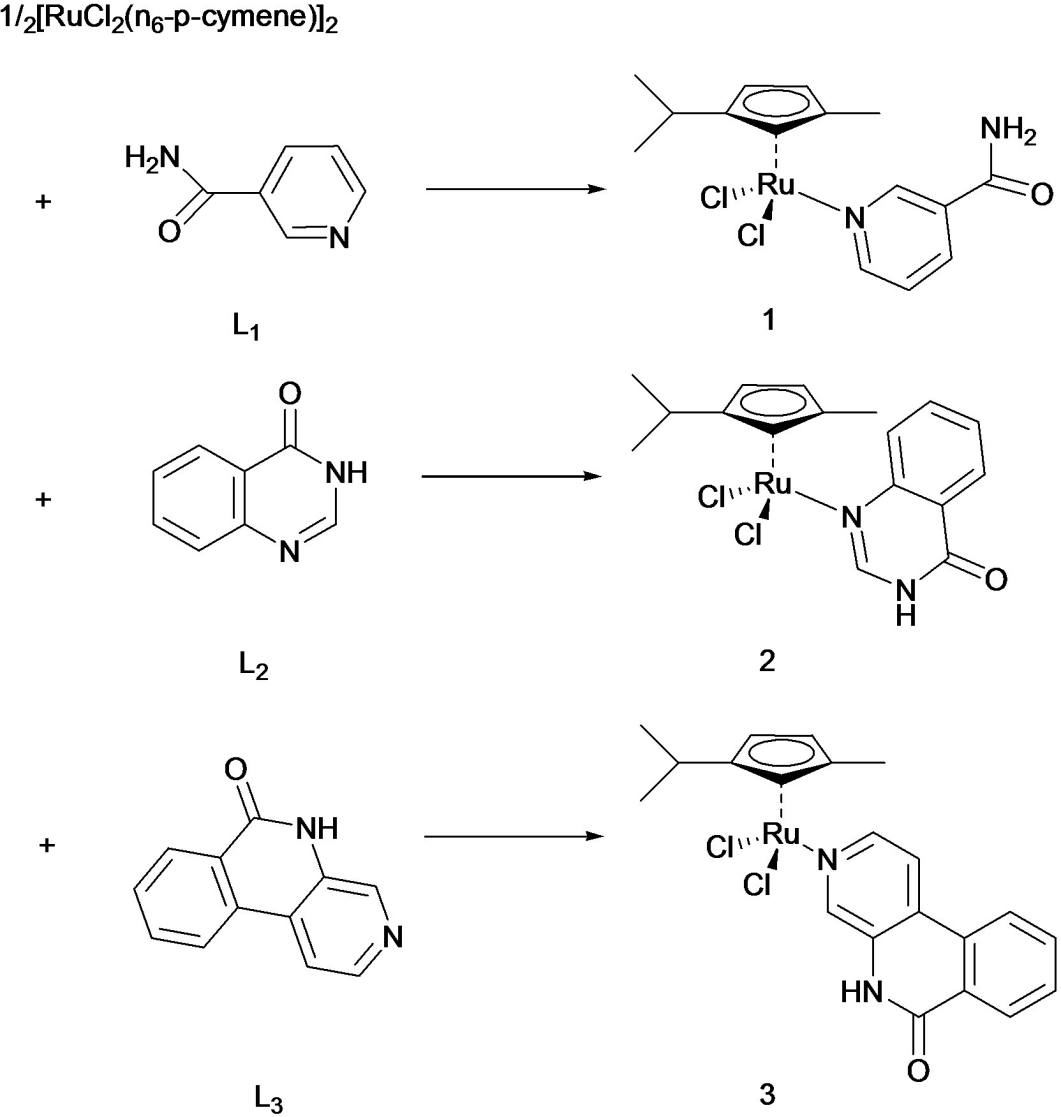
Structures of PARPi L1‐L3 (as the ruthenium coordinating ligand) and Ru‐PARPi 1–3 (conjugated ruthenium(II) arene complexes). Reprinted from Journal of Inorganic Biochemistry, 131, Wang, Z. et al., Multi‐targeted organometallic ruthenium(II)‐arene anticancer complexes bearing inhibitors of poly(ADP‐ribose) polymerase‐1: A strategy to improve cytotoxicity, 47–55, Copyright 2013, with permission from Elsevier.

Finally, Wang *et al*. suggested that the conjugation of ruthenium(II) arene moieties to PARPi significantly improved anti‐cancer activity in cancer cells *in vitro* compared to the reference compound RAPTA‐C (Figure 11).^[150]^ Anincrease in both cellular ruthenium content and cell‐cycle arrest at G1/S phase were observed following treatment with Ru‐PARPi. Compared to its free ligand, the coordination of PARPi to ruthenium also led to a more water‐soluble species. Further mechanistic investigations revealed that these ruthenium‐PARPi conjugates have improved PARP inhibitory activity compared to the free PARPi ligand and possess multi‐targeting properties through DNA binding and transcription inhibition. Encouragingly, a mild effect was observed in non‐malignant MRC5 human lung cells, likely due to the lower cellular uptake observed in these cells.

### 5.2 Gold complexes as PARP inhibitors

**Figure 12:**
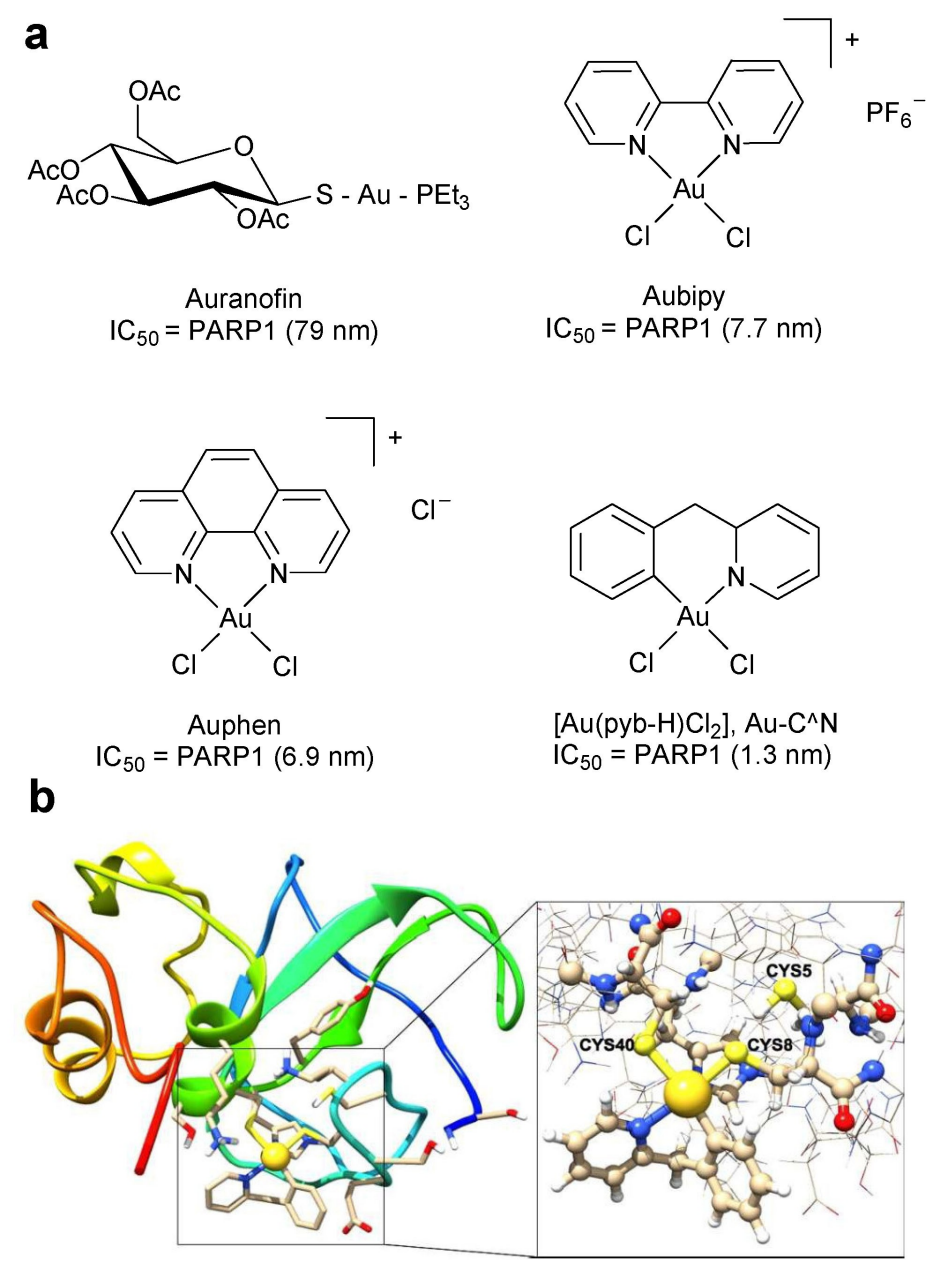
(a) Structures of antirheumatic agent, Auranofin and three Au(III) compounds (Aubipy, Auphen and Au−C−N). (b) Gold finger formation. Example of the possible binding of Au−C−N with the zinc finger domain of PARP1, in which two chlorido ligands of Au−C−N have been replaced by two cysteinato groups. Figure (b) adapted from reference 152 under the terms of the Creative Commons Attribution 3.0 Unported Licence (https://creativecommons.org/licenses/by/3.0/). Published by The Royal Society of Chemistry. © The Author 2018.

Strikingly, Mendes *et al*. also revealed the Au(I) antirheumatic agent auranofin (Au(2,3,4,6‐tetraO‐acetyl‐1‐(thio‐κS)‐β‐D‐glucopyranosato)PEt_3_ where PEt_3_=triethylphosphine) and two Au(III) polypyridyl complexes [Au(phen)Cl_2_]Cl and [Au(bipy)Cl_2_]Cl display low nanomolar IC_50_ values for PARP1 inhibition (Figure 12a).^[148]^

Decreased PARP1 activity is explained by the ability of the complexes to bind the zinc‐finger motif of PARP1, ejecting zinc and inactivating the enzyme in the process (Figure 12b).[^151^, ^152^] Although [Au(phen)Cl_2_]Cl, [Au(bipy)Cl_2_]Cl and derivatives display greater PARP1 inhibitory effects than olaparib and exhibit cytotoxicity in several cancer cell lines,^[153]^ their specificity towards BRCA‐deficient cancer cells or ability to synergise with DNA‐damaging agents or ionising radiation is unknown at present. Also of relevance is work by Citta *et al*., which described a PARP1 inhibitor coordinated to a Au(III) metal centre. In addition to showing antiproliferative effects against human cancer cells, the compound potently and selectively inhibits PARP1 with respect to the seleno‐enzyme thioredoxin reductase.^[154]^ Preliminary studies of this Au(III) PARPi indicated promising anti‐proliferative activity towards several cancer cell lines and an additive relationship when explored in combination with cisplatin.

## 6 Summary and Outlook

Typified by the platinum drugs and their potential successors in ruthenium complexes, many metal‐based complexes have been shown to kill cancer cells by interacting with DNA, resulting in the inhibition of DNA synthesis and/or generation of DNA damage. However, these single‐agent treatments are clinically limited, particularly in overcoming the ongoing challenge of drug resistance. The combinations of platinum‐based drugs and PARPi showed remarkable synergy in preclinical work and mechanistic findings revealed that these synergistic combinations are mainly associated with decreased DNA repair capacity and associated cell death from the accumulation of double‐strand break damage. Given the impressive clinical findings of combination therapies that demonstrated improved overall response rate with manageable toxicity profiles compared to each agent administered alone, these novel synergistic combination strategies have potential in improving clinical outcomes and achieving sufficient tumour suppression. While combination therapies of metal‐based complexes with DDR inhibitors are now being evaluated for synergy, further research is required to fully explore the underlying molecular mechanisms of synergy between compounds. With the expanding knowledge of the mechanisms of action of metallodrugs, combined with gained insight into drug synergy, rapid expansion in the studies on the combinations between metal‐based complexes with DNA repair inhibitors in clinical applications can be perceived. Although DNA‐targeting complexes hold the greatest promise to synergise with PARPi, it would be fascinating to examine whether mitochondrial‐targeting agents or PDT photosensitizers that generate cytotoxic levels of potentially DNA‐damaging reactive oxygen species (ROS) can likewise be combined with DDR inhibitors for additivity or synergy. Finally, the recent development of metal complexes inhibiting PARP activity opens up the possibility of designing dual‐function agents, combining both PARP inhibition and DNA‐binding (or ROS generation) in a single molecule.

## Conflict of interest

The authors declare no conflict of interest.

## Biographical Information

*Nur Aininie Yusoh received a BSc. in Biotechnology (2017) from Imperial College London. She then earned a master's degree in Medicinal Chemistry (2020) at the Department of Chemistry, Faculty of Science at the Universiti Putra Malaysia, under the supervision of Associate Professor Dr. Haslina Ahmad. Her thesis in medicinal chemistry focused on the combination of a ruthenium polypyridyl complex and PARP inhibitors for cancer treatment. Her research interests include drug design, synthesis and in vitro/in vivo biological evaluation assays*.

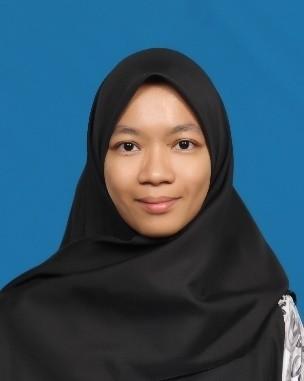

## Biographical Information

*Haslina Ahmad is a synthetic chemist who has been active in drug design, drug delivery and nanomaterial synthesis for almost 11 years. She received her PhD in Bioinorganic Chemistry (2009) from the University of Sheffield. She has started her academic position at the Universiti Putra Malaysia (2009), and currently an Associate Professor in Inorganic Chemistry. Her research focus includes design, synthesis and drug delivery of metal‐based compounds for cancer. She is the author of over 30 publications (citations: 318, H‐index:12) and 60 conference presentations*.

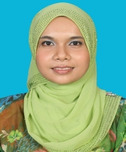

## Biographical Information

*Martin Gill obtained his PhD in Chemistry from the University of Sheffield in 2010. After EPSRC and Wellcome Trust Fellowships at the University of Sheffield, Martin continued his postdoctoral research at the Oxford Institute for Radiation Oncology, University of Oxford. Since 2019, Martin has been a Lecturer in Chemistry in the Department of Chemistry at Swansea University in his native Wales. His research mixes inorganic chemistry with cell biology to explore the medicinal chemistry and chemical biology of transition metal complexes*.